# Beliefs, Barriers, and Stretching Practices Among Recreational Snowboarders and Alpine Skiers: A Cross-Sectional Study with a Generational Perspective

**DOI:** 10.3390/sports14020055

**Published:** 2026-02-03

**Authors:** Julio Camacho, María B. García-Moreno, Francisco Requena, Rocío Camacho, Manuel Pérez-Priego, Estrella I. Agüera

**Affiliations:** 1 Cellular Biology, Physiology and Immunology Department, Campus of Rabanales, University of Cordoba, 14071 Cordoba, Spain; z32caagj@uco.es (J.C.); m92caagr@uco.es (R.C.);; 2 Statistics and Econometrics Department, Agri-Food Campus of International Excellence ceiA3, University of Cordoba, 14071 Cordoba, Spain; d52gagam@uco.es (M.B.G.-M.); mppriego@uco.es (M.P.-P.)

**Keywords:** flexibility, injury prevention, outdoor sports, exercise adherence, digital health information

## Abstract

Stretching is commonly recommended to improve flexibility and reduce injury risk; however, its practical implementation among recreational snowboarders and alpine skiers remains inconsistent. A cross-sectional online survey was conducted during the 2024–2025 winter season. Of 403 collected responses, 391 valid questionnaires were included after data screening. The survey assessed sociodemographic characteristics, stretching perceptions, practices, perceived barriers, information sources, and supervision. Descriptive statistics and chi-square tests were used to explore associations between sport modality and generational cohorts. Although 91.3% of participants considered stretching necessary, only 39.7% reported performing stretching exercises in the previous six months. The most frequently reported barriers were lack of time (76.1%) and low motivation (54.2%). Alpine skiers attributed greater importance to stretching than snowboarders. Significant generational differences were observed in sport modality, practice volume, and information-seeking behaviour (*p* < 0.05), but not in stretching frequency or duration; therefore, Hypothesis 1 was not supported. Only 22.7% of participants reported receiving professional supervision. In recreational winter sports, stretching is widely valued but inconsistently practiced. Behaviour appears to be driven more by motivational and contextual factors than by generational differences in practice frequency, highlighting the need for targeted educational strategies and evidence-informed guidance.

## 1. Introduction

Stretching is commonly incorporated into sport and exercise routines and is traditionally understood as a practice aimed at lengthening muscle–tendon units to improve flexibility and prepare the body for physical activity [1,2]. Over recent decades, research has clearly demonstrated that stretching is not a homogeneous intervention but rather encompasses a wide range of techniques that may differ substantially in their mechanical, neuromuscular, and perceptual effects [3,4,5,6]. These techniques include static stretching, dynamic stretching, ballistic stretching, proprioceptive neuromuscular facilitation, and combined approaches, each of which may influence range of motion (ROM) through different mechanisms and with varying magnitudes [7,8,9].

Accumulating evidence indicates that different stretching modalities can improve ROM via distinct physiological pathways. Meta-analytical and narrative syntheses have shown that chronic static stretching primarily increases ROM through enhanced stretch tolerance rather than through structural changes in muscle–tendon properties, whereas dynamic stretching may elicit ROM gains through neuromuscular activation and coordination mechanisms [3,6]. Moreover, recent experimental studies have highlighted the potential benefits of combining stretching with adjunct techniques. For example, a five-week intervention integrating foam rolling with static stretching resulted in greater improvements in ankle joint ROM, angular velocity, and countermovement jump performance compared with static stretching alone [10]. Together, these findings underscore that stretching outcomes depend not only on whether stretching is performed, but also on the specific type and combination of techniques applied.

Despite the extensive body of research, there is currently no consensus regarding optimal stretching prescriptions. Recent reviews and expert consensus statements emphasize substantial uncertainty not only about the most effective stretching dose, but also about which stretching modalities are most appropriate for specific populations, contexts, or performance objectives [11,12]. This lack of agreement is particularly relevant in recreational sport settings, where stretching practices are rarely guided by standardized protocols and are instead shaped by personal experience, informal advice, and perceived benefits [9,13,14].

Recreational alpine skiing and snowboarding constitute physically demanding activities characterized by intermittent exposure, high mechanical loads, and variable environmental conditions. However, stretching behaviours in these populations remain under-researched compared with other sports [15,16,17,18]. Moreover, recreational winter-sport participants span a wide age range and may differ markedly in their training background, access to professional guidance, and sources of information related to physical preparation and injury prevention [19,20,21]. In such contexts, stretching practices may reflect motivational factors, perceived barriers, and information-seeking behaviours as much as, or more than, evidence-based recommendations.

These cohorts differ not only in chronological age but also in shared sociocultural experiences, health beliefs, digital engagement, and patterns of risk perception, all of which may shape how recreational athletes approach physical preparation and injury-prevention strategies.

Beyond sport-specific factors, there is growing evidence that generational influences shape health-related decisions, including engagement in injury-prevention practices such as stretching. Generational differences in lifestyle, value priorities, digital fluency, and access to health information have been shown to affect exercise behaviour and risk-management strategies [21,22,23]. To account for these influences, the present study adopts the generational framework originally proposed by Strauss and Howe [21], as subsequently applied and adapted in behavioural and health-related research [22,23]. Accordingly, four generational cohorts are considered: Baby Boomers (1943–1960), Generation X (1961–1981), Millennials or Generation Y (1982–1994), and Generation Z (1995–2010).

From a behavioural perspective, applying a generational framework is particularly relevant for understanding stretching behaviour, as preventive practices are strongly influenced by sociocultural norms, formative experiences, and health-related values acquired across the life course. Generational cohorts have been shown to exhibit distinct psychosocial and health-behavioural trajectories, suggesting that generational membership may be associated with variability in motivation, perceived barriers, and adherence to preventive routines.

Nevertheless, empirical evidence examining stretching habits and perceptions across generational cohorts remains scarce, particularly in recreational winter sports, and existing studies have largely focused on other populations or professional athletes. Accordingly, the aim of the present study was to explore stretching habits, perceptions, and perceived barriers among recreational alpine skiers and snowboarders, with particular attention to potential differences across generational cohorts. Given the observational, cross-sectional design and the ongoing lack of consensus regarding optimal stretching modalities and prescriptions, this study is explicitly positioned as exploratory, seeking to describe real-world behavioural patterns rather than to evaluate the effectiveness of specific stretching interventions.

Based on the literature and theoretical frameworks described above, the following hypotheses were formulated:

**H1.**  *The frequency of stretching practices differs across generational cohorts, reflecting variations in health-related behaviours, motivation, and engagement in preventive strategies.*

**H2.**  *The perceived importance of stretching differs between alpine skiers and snowboarders, with alpine skiers expected to assign greater importance due to sport-specific biomechanical demands and injury-risk profiles.*

**H3.**  *Perceived barriers to stretching—such as lack of time, low motivation, and insufficient knowledge—differ across generational cohorts, in line with generational differences in health beliefs and decision-making patterns.*

## 2. Materials and Methods

### 2.1. Study Design

A cross-sectional observational study was conducted using an online survey (SurveyMonkey, San Mateo, CA, USA) to examine stretching-related perceptions, practices, and associated factors among recreational snowboarders and alpine skiers. The study followed a descriptive and exploratory design, aimed at characterizing patterns and associations within the sampled population rather than testing causal relationships. Data were collected during the 2024–2025 winter season, corresponding to the active period of alpine skiing and snowboarding practice.

### 2.2. Participants and Recruitment

Participants were recruited using a non-probabilistic, self-selected sampling strategy. The survey link was disseminated through open online channels, including social media platforms and email distribution, and was accessible to recreational snowboarders and alpine skiers aged 18 years or older. Participation was voluntary and anonymous, and no incentives were offered.

Because the survey was disseminated through open online channels, the total number of individuals exposed to the survey link could not be quantified.

### 2.3. Ethical Considerations

The study was carried out in accordance with the principles of the Declaration of Helsinki and was approved by the Bioethics Committee of the University of Cordoba, Spain (approval code: CEIH_24-17). All participants were informed about the purpose of the study, the voluntary nature of participation, and the anonymous handling of data prior to providing informed consent. No personally identifiable information was collected.

### 2.4. Questionnaire and Measures

The survey instrument comprised 20 closed-ended questions, some of which allowed multiple responses in order to capture the non–mutually exclusive nature of several constructs. The questionnaire was structured into four clearly differentiated sections, and conditional logic was implemented so that respondents were automatically directed to specific questions based on their previous answers, ensuring the relevance of each section to individual participant profiles.

Section 1 collected socio-demographic and sport-related information, including gender, educational level, geographical origin, primary sport practiced (alpine skiing, snowboarding, or both), and self-reported skill level. Sport-related variables included the primary sport practiced, weekly time devoted to sport practice, and self-perceived technical proficiency.

Participants’ skill levels were categorized according to a classification system adapted from Luppino et al. [24] for recreational skiing and snowboarding. Four levels were defined—beginner, intermediate, advanced, and expert—based on participants’ self-reported ability to manage different slopes, terrains, and snow conditions. This classification was adopted to promote consistency in the assessment of skill levels and to facilitate the interpretation of results in relation to skill-specific factors.

Section 2 focused on participants’ perceptions regarding the need to perform stretching exercises in the context of alpine skiing and snowboarding, including motivations and perceived appropriate timing of stretching.

Section 3 examined participants’ self-reported stretching behaviours during the previous six months, including stretching frequency, typical session duration, targeted body regions, and reasons for performing or not performing stretching exercises.

Section 4 investigated information-seeking behaviours and supervision related to stretching practices, including the sources of information consulted and whether stretching was performed under professional supervision.

The questionnaire was developed based on previous literature and expert input to ensure content relevance and clarity for recreational winter-sport participants. Given the exploratory nature of the study and the absence of validated instruments specifically designed to assess stretching practices in this population, the questionnaire was intended to capture real-world behaviours and perceptions rather than to function as a psychometrically validated scale. Responses were recorded using categorical options and Likert-type scales, enabling descriptive and comparative analyses across subgroups. To enhance transparency and reproducibility, sample questions from the questionnaire are provided in Supplementary File S1 (Supplementary Materials).

### 2.5. Instrument Development and Content Validation

The questionnaire was adapted from instruments previously used to examine stretching practices and perceptions in recreational and sport contexts [25]. Minor wording adjustments were made to ensure clarity and relevance to alpine skiing and snowboarding populations.

The instrument underwent a three-stage content validation process prior to its implementation. First, a qualitative review was conducted by a panel of experts composed of coaches and instructors specialized in winter sports, who evaluated the relevance, clarity, and adequacy of the items after being informed about the objectives of the study. In the second stage, the questionnaire was reviewed by a group of federated athletes to assess its suitability and comprehensibility within the specific context of recreational alpine skiing and snowboarding. Finally, a pilot study was conducted with a randomly selected sample of 20 athletes who met the study inclusion criteria, with the aim of identifying potential ambiguities, comprehension difficulties, or feasibility issues prior to large-scale data collection.

### 2.6. Data Handling and Variable Treatment

Prior to analysis, data were screened for completeness and internal consistency. Responses with substantial missing data were excluded. For variables allowing multiple response options, each option was treated as a binary indicator (selected/not selected) for descriptive and inferential purposes. Categories with very low frequencies were collapsed where appropriate to satisfy analytical assumptions. With regard to data screening and response validity, responses were considered valid if participants met the inclusion criteria (≥18 years of age; recreational alpine skiing and/or snowboarding) and completed all core sections required for analysis (Sections 1–4 of the questionnaire). Responses with incomplete data in any of these core sections were excluded during the screening process (*n* = 12). After data screening, a total of 391 valid responses were retained for descriptive and inferential analyses.

Due to insufficient expected cell frequencies, participants classified as Baby Boomers were excluded from inferential analyses involving generational comparisons.

### 2.7. Statistical Analysis

Statistical analyses were performed using appropriate software (IBM SPSS Statistics, version 29; IBM Corp., Armonk, NY, USA). Given the exploratory nature of the study, the cross-sectional design, and the predominantly categorical structure of the variables, the analysis focused on descriptive statistics and bivariate association testing.

Categorical variables are presented as absolute frequencies and percentages. Descriptive analyses were conducted to summarize socio-demographic characteristics, sport-related variables, stretching practices, perceptions regarding the need for stretching, perceived barriers, and information-seeking behaviours.

Associations between categorical variables were examined using Pearson’s chi-square (χ^2^) test. When expected cell counts were insufficient to meet χ^2^ assumptions, appropriate corrections or alternative exact tests were applied as required. The level of statistical significance was set at *p* < 0.05.

Given the non-probabilistic sampling strategy, the imbalance across generational cohorts, and the absence of an a priori power calculation, no multivariable modelling or causal inference was attempted. Accordingly, the results are interpreted as descriptive and exploratory, intended to identify association patterns rather than to establish causal relationships.

### 2.8. Study Flowchart

Table 1 summarizes the flow of participants through recruitment, eligibility screening, and inclusion in descriptive and inferential analyses.

## 3. Results

### 3.1. Sociodemographic Characteristics of the Participants

The sociodemographic characteristics of the participants are presented in Table 2. The final descriptive sample consisted of 391 recreational snowboarders and alpine skiers. Regarding gender, 44.2% of participants were male (*n* = 173), 50.1% were female *(n* = 196) and 5.6% identified as non-binary (*n* = 22).

With respect to educational level, the highest proportion of participants reported university education (58.3%), followed by secondary education (33.8%) and 7.9% of participants had primary education. Participants were distributed across generational cohorts as follows: Generation Y (Millennials) represented the largest proportion of the sample (54.8%, *n* = 214), followed by Generation Z (25.7%, *n* = 100), Generation X (18.8%, *n* = 74), and Baby Boomers (0.8%, *n* = 3).

Most participants reported Spain as their country of origin (88.5%), while smaller proportions indicated other European countries (8.9%) or non-European countries (2.6%).

### 3.2. Sport Modality and Self-Reported Skill Level

The distribution of sport modality according to self-reported skill level is shown in Table 3. Across the total sample, participants reported practicing snowboarding, alpine skiing or both modalities.

Among participants classified as beginners, snowboarding was reported by 24.8%, alpine skiing by 58.9%, and both modalities by 16.3%. In the intermediate group, the corresponding proportions were 37.5% reported by snowboarders, 32.3% by intermediate, and both by 30.2%. Among advanced participants, 21.8% reported snowboarding, 46.6% reported alpine skiing, and 31.1% reported practicing both modalities. Among expert participants, 27.6% practiced snowboarding only (*n* = 20), 42.1% practiced alpine skiing only (*n* = 31), and 30.3% reported practicing both modalities (*n* = 22).

Exploratory χ^2^ analysis indicated a statistically significant association between sport modality and self-reported skill level (χ^2^ test; Table 3).

### 3.3. Weekly Practice Time According to Self-Reported Skill Level

Weekly practice time stratified by self-reported skill level is presented in Table 4. In the total sample, 5.0% of participants (*n* = 20) reported practicing less than 2 h per week, 31.9% of participants (*n* = 124) reported practicing between 3 and 6 h per week, 30.9% (*n* = 121) between 7 and 10 h per week, and 32.2% more than 10 h per week (*n* = 126). Additional descriptive information regarding weekly stretching frequency, session duration, and body regions targeted is provided in Figure S1 (Supplementary File S2).

Weekly practice time showed a clear distributional pattern across self-reported skill levels. Among beginners, the most frequently reported category was 3–6 h per week (82.7%). In the intermediate group, the modal category shifted to 7–10 h per week (67.7%). In both the Advanced and expert groups, the most frequently reported category was more than 10 h per week (55.3%) and (84.2%) respectively. These results describe the observed exposure profiles within the sampled population.

The χ^2^ test revealed a statistically significant association between weekly practice time and self-reported skill level (χ^2^ = 361.62; *p* < 0.001; Table 4).

As preliminary considerations informing the analysis of more specific aspects presented in subsequent sections, the general responses to key questions were first examined. Of the total valid sample (391), 357 participants (91.3%) indicated that stretching exercises should be performed. These respondents subsequently answered questions regarding their perception of stretching practice and the underlying motivations for this belief. However, a discrepancy was observed between perception and actual behaviour: only 142 participants (39.7%) reported having performed stretching exercises within the last six months, while 215 participants (60.3%)—despite acknowledging the importance of stretching—had not engaged in this practice during the same period. The motivations reported by participants in both groups constitute the basis for the results on stretching as a habitual practice, including the characteristics of the exercises performed by those who do engage in them. Additionally, 220 participants (56.2%) reported having consulted information at some point to understand or perform stretching exercises, and the most frequently used sources were identified. Similarly, 89 participants (22.7%) indicated having received supervision while performing these exercises, and the professional profile of the supervisors most commonly consulted was analyzed.

### 3.4. Perceived Importance of Stretching and Underlying Motivations

Following the description of the respondents’ sociodemographic profile, the analysis focused on perceptions regarding the importance of stretching exercises and the motivations underlying these beliefs. Overall, 357 participants (91.3%) reported that stretching exercises should be performed and indicated one or more reasons supporting this view.

**Figure 1:**
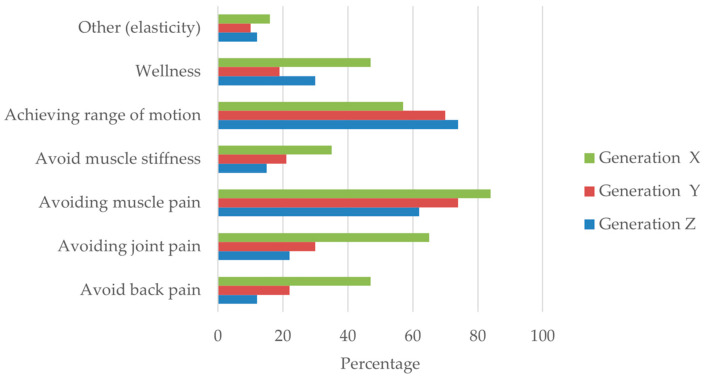
Reported motivations for endorsing stretching among respondents who indicated that stretching should be performed (*n* = 357). Bars represent the proportion of participants within each generational cohort (Generations X, Y, and Z) who selected each motivation. Multiple responses were allowed; therefore, proportions do not sum to 100%. Baby Boomers were excluded from stratified displays due to the very small sample size.

As shown in Figure 1, the most frequently reported motivations across the sample were the prevention of muscle pain and the improvement of range of motion. Other motivations, reported by a smaller proportion of participants, included avoiding joint pain, preventing back pain, reducing muscle stiffness, and promoting general well-being. These responses reflect a predominantly preventive and functional perception of stretching among recreational alpine skiers and snowboarders.

When the data were examined by generational cohort, distinct motivational patterns were observed. Participants from Generation X more frequently reported pain-related motivations, particularly the prevention of muscle, joint, and back pain. In contrast, Generations Y and Z showed similar motivational profiles, primarily emphasizing muscle pain prevention and improvements in range of motion, with comparatively lower endorsement of well-being-related motivations. Due to the very small number of Baby Boomers, this cohort was excluded from stratified visual displays.

Perceptions regarding the necessity of performing stretching exercises also varied according to the type of sport practiced. A higher proportion of alpine skiers reported considering stretching necessary, whereas this perception was less frequent among snowboarders. Participants who practiced both alpine skiing and snowboarding showed an intermediate pattern, with a substantial proportion acknowledging the relevance of stretching exercises.

Multiple responses were allowed for this item. Absolute frequencies represent the number of selections rather than the number of participants; therefore, totals may exceed the size of the corresponding subsample.

### 3.5. Reported Barriers to Performing Stretching Exercises

The analysis of perceived barriers focused on participants who reported that stretching exercises should be performed but did not engage in this practice. Within this subgroup (*n* = 215), several barriers were commonly reported, reflecting practical and motivational constraints.

**Figure 2:**
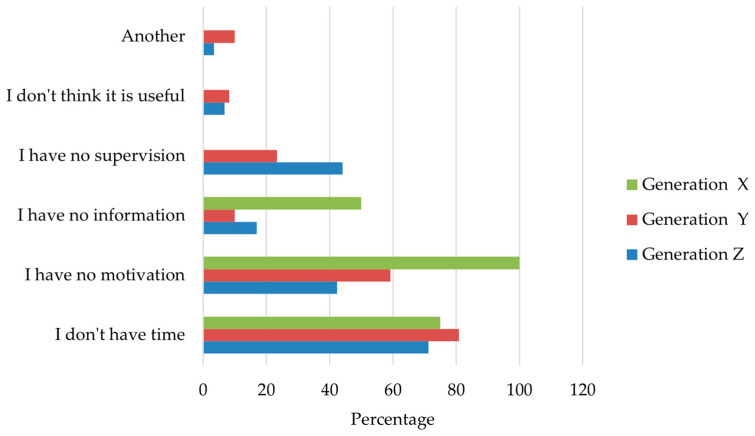
Reported barriers to performing stretching among respondents who indicated that stretching should be performed (*n* = 357). Bars represent the proportion of participants within each generational cohort (Generations X, Y, and Z) selecting each barrier. Multiple responses were allowed; therefore, proportions do not sum to 100%. Baby Boomers were excluded from stratified displays due to the very small sample size.

As illustrated in Figure 2 the most frequently cited barriers across the sample were lack of time and lack of motivation. A substantial proportion of participants also reported the absence of professional supervision as a limiting factor. Other barriers, such as insufficient information about stretching exercises or the perception that stretching is not useful, were reported less frequently.

When examined by generational cohort, differences in the relative prominence of specific barriers were observed. Lack of motivation was commonly reported across Generation X and Generation Y, whereas lack of time was particularly frequent among participants from Generations Y and Z. Barriers related to insufficient information and lack of supervision were less prevalent overall but showed a higher relative representation in the oldest cohort. Due to the very small number of Baby Boomers, this group was excluded from stratified visual displays.

### 3.6. Information Sources and Supervision in Stretching Practice

This section examines the sources of information consulted by participants to learn about stretching exercises, as well as the presence of professional supervision during stretching practice. Participants who reported having consulted information about stretching exercises (*n* = 219) were allowed to select multiple sources. Therefore, the absolute frequencies shown for individual information sources represent the number of selections rather than the number of participants, and totals may exceed the size of the subsample.

**Figure 3:**
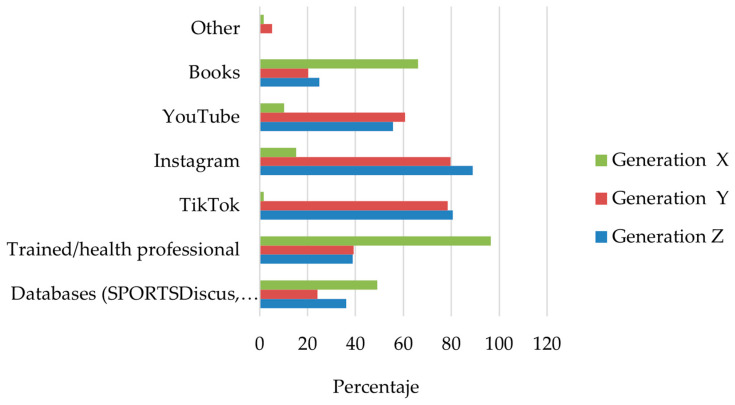
Sources of information consulted to learn about stretching among respondents who reported seeking information on this topic (*n* = 219). Bars represent the proportion of participants within each generational cohort (Generations X, Y, and Z) selecting each information source. Multiple responses were allowed; therefore, proportions do not sum to 100%. Baby Boomers were excluded from stratified displays due to the very small sample size.

As shown in Figure 3, social media platforms played a prominent role as sources of information across the sample. Instagram and TikTok were among the most frequently mentioned channels, followed by video-based platforms such as YouTube. In addition to these digital sources, a substantial proportion of participants reported obtaining information from more formal sources, including coaches, instructors, or health professionals. The relative prominence of specific information sources varied across generational cohorts, with younger participants reporting greater reliance on social media–based platforms.

**Figure 4:**
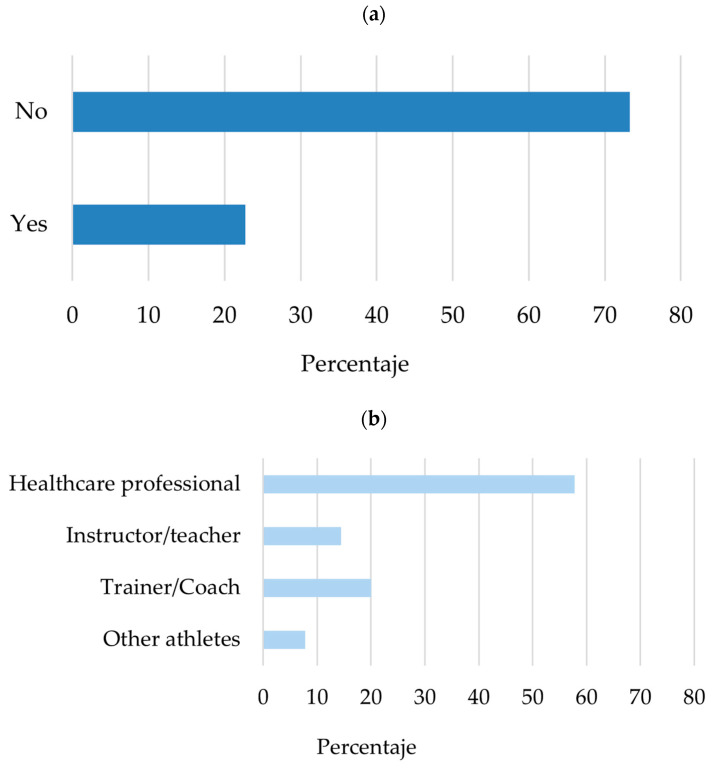
Supervision of stretching practice among recreational alpine skiers and snowboarders: (**a**) proportion of participants reporting supervised versus unsupervised stretching (*n* = 391) and (**b**) type of professional providing supervision among those reporting supervised stretching (*n* = 89). Multiple responses were allowed in panel (**b**); therefore, proportions do not sum to 100%.

In addition to information seeking, supervision during stretching practice was also examined. As illustrated in Figure 4, only a minority of participants reported performing stretching exercises under professional supervision, whereas most indicated that their stretching routines were unsupervised. Among those who reported receiving supervision, guidance was most commonly provided by coaches or instructors and by health professionals.

### 3.7. Analysis of Dependence Between the Generation Variable and the Other Variables

A series of exploratory Pearson’s χ^2^ tests was conducted to examine associations between generational cohort and the variables included in the descriptive analyses (Table 5). To satisfy χ^2^ test assumptions and avoid unreliable estimates due to sparse cell counts, participants classified as Baby Boomers were excluded from inferential analyses; therefore, results refer exclusively to Generations X, Y (Millennials), and Z.

## 4. Discussion

The present cross-sectional study provides an exploratory characterization of stretching habits, perceptions, and perceived barriers among recreational alpine skiers and snowboarders. Overall, the findings indicate that stretching is widely perceived as relevant; however, its practical implementation is inconsistent and appears to be influenced more by motivational and contextual factors than by clear differences in practice frequency across generational cohorts. This pattern is consistent with previous survey-based research reporting a discrepancy between positive attitudes toward stretching and its actual adoption in recreationally active populations [25].

### 4.1. Stretching Perceptions in a Context of Heterogeneous Evidence

Stretching is often communicated as a generic and universally beneficial practice, despite the substantial heterogeneity of stretching modalities and outcomes reported in the literature [3,4,5,6,7,8]. Recent scoping reviews and expert consensus statements emphasize that there is no single optimal stretching protocol applicable across populations, contexts, or objectives [11,12]. In this context, it is not surprising that recreational athletes develop beliefs about stretching that may not fully reflect the complexity or uncertainty of the scientific evidence. The present findings suggest that perceptions of stretching importance may coexist with limited or irregular practice, reinforcing the need to examine stretching not only as a physiological intervention but also as a behavioural phenomenon shaped by simplified messages and contextual constraints.

### 4.2. Stretching Frequency and Hypothesis Testing

A central finding of this study is the absence of significant differences in stretching frequency across generational cohorts. Accordingly, Hypothesis H1, as originally formulated, was not supported by the data. This result indicates that generational cohort membership, at least within the limitations of the present sample, does not explain variability in stretching frequency among recreational alpine skiers and snowboarders. Importantly, this finding underscores the need to distinguish between chronological age and generational cohort effects. While generational frameworks may capture shared sociocultural experiences [21], they are inherently confounded with age- and life-stage–related factors, which cannot be disentangled in a cross-sectional design [19,26]. Therefore, any generational interpretation should be regarded as exploratory rather than confirmatory.

### 4.3. Barriers and Motivational Determinants

Although stretching frequency did not differ by cohort, participants reported several barriers that plausibly limit implementation, including lack of time, reduced motivation, and insufficient knowledge. These barriers are consistent with broader models of health behaviour and physical activity adherence, which emphasize that perceived benefits alone are insufficient to ensure consistent behaviour [26,27]. In recreational winter sports, participation is often episodic and constrained by environmental and logistical factors, which may further reduce the priority given to preparatory routines such as stretching. Importantly, the associations observed in this study are descriptive and should not be interpreted causally, as the cross-sectional design precludes inference regarding directionality or mechanism.

### 4.4. Information Sources and Unsupervised Practice

The reliance on informal or digital sources of information reported by a substantial proportion of participants aligns with contemporary patterns of health information seeking [28]. While digital platforms can facilitate access to exercise-related content, they also expose users to information of variable quality, including misinformation [29]. In the absence of professional supervision, such exposure may contribute to simplified or inconsistent stretching practices. However, the present study did not assess the accuracy or quality of the information consumed, and these findings should therefore be interpreted as contextual indicators rather than evaluative conclusions.

### 4.5. Methodological Limitations and Interpretative Scope

Importantly, the limitations of the present study should be regarded as critical constraints on generalizability, rather than as minor methodological considerations. The use of a non-probabilistic, self-selected sampling strategy introduces a high risk of selection bias, likely overrepresenting individuals who are more health-conscious and digitally engaged than the broader population of recreational alpine skiers and snowboarders, thereby substantially limiting external validity. In addition, the cross-sectional design precludes temporal or causal inference, and the absence of an a priori sample size or power calculation reduces confidence in detecting small effects, particularly in subgroup analyses.

Furthermore, the marked imbalance in generational representation—most notably the minimal inclusion of Baby Boomers—precludes robust generational comparisons and restricts interpretability. Although the questionnaire was adapted from previously used instruments, the lack of formal validation across age groups raises the possibility of age-related measurement bias. Internal consistency indices (e.g., Cronbach’s alpha) were not calculated, as the questionnaire was not intended to function as a psychometric scale but rather as a descriptive survey tool designed for exploratory purposes. Finally, the reliance on bivariate analyses without multivariable adjustment leaves residual confounding unaddressed, and the use of multiple comparisons may increase the risk of type I error.

Taken together, these limitations require that the findings be interpreted strictly as exploratory and preliminary, intended to describe association patterns within the sampled population and to inform the design of future, methodologically robust studies, rather than to support confirmatory or generalizable conclusions.

### 4.6. Contribution and Implications for Future Research

Despite these limitations, the study contributes novel exploratory insights into stretching-related behaviours in an under-researched recreational winter-sport context. By focusing on perceptions, barriers, and self-reported practices rather than on physiological efficacy (which remains heterogeneous and context-dependent [11,12], and has been shown to vary according to stretching modality and combination strategies [10]) the present findings complement experimental research and highlight the importance of behavioural determinants in real-world settings. Future studies should employ representative sampling strategies, validated instruments across age groups, and multivariable analytical approaches to clarify the determinants of stretching behaviour and to inform evidence-aligned educational interventions. As such, the present findings should be viewed as a descriptive starting point rather than as evidence for cohort-specific behavioural differences.

## 5. Conclusions

This cross-sectional exploratory study indicates that, among recreational alpine skiers and snowboarders, stretching is generally perceived as important, yet its implementation is inconsistent. Importantly, no significant generational differences in stretching frequency were observed; therefore, Hypothesis H1, as originally formulated, was not supported by the data.

The absence of generational differences in stretching frequency should be interpreted with caution. While generational cohort membership may reflect shared sociocultural experiences, chronological age is more closely related to biological and life-stage factors. Given the cross-sectional design of the present study, these dimensions cannot be disentangled, and the findings do not allow attribution of observed patterns to cohort-specific effects rather than to age-related or contextual influences.

Overall, stretching behaviour in this recreational winter-sport context appears to be more closely associated with motivational and contextual barriers, such as limited time, reduced motivation, and insufficient knowledge, than with generational differences in practice frequency. However, due to the observational nature of the study, these associations should not be interpreted causally.

In light of the non-probabilistic, self-selected sample, cohort imbalance, absence of an a priori power calculation, and potential measurement limitations, the findings should be regarded as exploratory and preliminary and interpreted with caution. They cannot be generalized beyond populations with characteristics similar to those of the study sample. Nevertheless, the study provides a transparent exploratory overview of stretching-related perceptions and practices in recreational winter sports and offers a foundation for future research employing representative sampling, validated instruments across age groups, and multivariable analytical approaches.

## Figures and Tables

**Table 1:** Flow of participants through the study. *n* indicates the number of participants at each study stage. For the recruitment stage, the total number of individuals exposed to the survey link could not be determined due to the open online dissemination strategy. NA (not applicable).

Study Stage	Description	*n*
Recruitment	Survey link disseminated via social media platforms and email during the 2024–2025 winter season.	NA
Initial responses	Completed survey responses received.	403
Eligibility screening	Responses excluded due to incomplete data or failure to meet inclusion criteria.	12
Descriptive sample	Valid responses included in descriptive analyses	391
Inferential screening	Participants excluded from inferential analyses due to insufficient expected cell frequencies (Baby Boomers).	3

**Table 2:** Sociodemographic characteristics of the participants, including gender, educational level, generational cohort, and geographical origin.

Variables	Categories	Total
Gender	Male	44.2% (173)
	Female	50.1% (196)
	Non-binary	5.6% (22)
Level of education	Primary education	7.9% (31)
	Secondary education	33.8% (132)
	University	58.3% (228)
Generational cohort	Baby Boomer	0.8% (3)
	Generation X	18.8% (74)
	Generation Y (millennials)	54.8% (214)
	Generation Z (centennials)	25.7% (100)
Geographical Origin	National (Spain)	88.5% (348)
	European countries	8.9% (35)
	Non-European countries	2.6% (10)

Data presented as *n* (%). Percentages may not total 100% due to rounding.

**Table 3:** Distribution of sport modality according to self-reported skill level among recreational alpine skiers and snowboarders (χ^2^ test).

Sport Modality	Total *n* (%)	Beginner *n* = 125 (31.9%)	Intermediate *n* = 93 (23.8%)	Advanced *n* = 100 (25.5%)	Expert *n* = 73 (18.8%)
Snowboarding	108 (32.2%)	31 (24.8%)	35 (37.5%)	22 (21.8%)	20 (27.6%)
Alpine skiing	182 (46.8%)	74 (58.9%)	30 (32.3%)	47 (46.6%)	31 (42.1%)
Both	101 (21%)	20 (16.3%)	28 (30.2%)	31 (31.1%)	22 (30.3%)

Data presented as *n* (%). Pearson’s χ^2^ test was used to examine the association between sport modality and self-reported skill level. Results should be interpreted as exploratory.

**Table 4:** Weekly practice time according to self-reported skill level among recreational snowboarders and alpine skiers (χ^2^ test).

Weekly Practice Time	Total *n* (%)	Beginner *n* = 125 (31.9%)	Intermediate *n* = 93 (23.8%)	Advanced *n* = 100 (25.5%)	Expert *n* = 73 (18.8%)
<2 h	20 (5.0%)	3 (2.3%)	8 (8.3%)	7 (6.8%)	2 (2.6%)
3–6 h	124 (31.9%)	103 (82.2%)	15 (16.7%)	3 (3.3%)	3 (3.9%)
7–10 h	121 (30.9)	17 (14%)	63 (67.7%)	34 (34%)	7 (9.2%)
>10 h	126 (32.2%)	2 (1.6%)	7 (7.3%)	56 (55.3%)	61 (84.2%)

Data presented as *n* (%). Pearson’s χ^2^ test indicated a statistically significant association between weekly practice time and self-reported skill level (χ^2^ = 361.62; *p* < 0.001).

**Table 5:** Results of the Chi-Square test to analyze the dependence between the generation variable and the other variables studied. The Baby Boomer cohort was excluded from inferential analyses due to insufficient expected frequencies; results therefore refer to generations X, Y, and Z only.

Variables	Χ^2^	*p*-Value
Sport practised	105.180	<0.000 *
Level	81.750	<0.000 *
Hours per week spent on these sports	71.787	<0.000 *
Belief in the need for stretching exercises	9.226	0.010 *
Frequency with which stretching exercises are performed	11.813	0.160
Duration of stretching exercises	1.881	0.390
Part of the body worked	3.71	0.447
Performing stretching exercises during the last 6 months	0.574	<0.000 *
Consultation of information to help understand and perform stretching exercises	37.978	<0.000 *

* Variable showing statistically significant differences in frequency distribution between different generations at a significance level of 5%, indicating dependence between the two.

## Data Availability

The data presented in this study are available on request from the corresponding author due to this study are part of an ongoing doctoral thesis and are therefore not publicly available at this stage.

## Supplementary Materials

The following supporting information can be downloaded at: https://www.mdpi.com/article/10.3390/sports14020055/s1, Supplementary File S1 contains sample questions from the questionnaire used in this study (PDF). Supplementary File S2 contains Figure S1 showing descriptive characteristics of habitual stretching practices among recreational alpine skiers and snowboarders (PDF).
