# Supplementary material for: Beliefs, Barriers, and Stretching Practices Among Recreational Snowboarders and Alpine Skiers: A Cross-Sectional Study with a Generational Perspective
Source: Sports (Basel). 2026 Feb 3;14(2):55. doi: 10.3390/sports14020055 (PMC12944958; doi:10.3390/sports14020055)
Supplement: Supplementary file 1 [file sports-14-00055-s001.zip › Supplementary File S1.pdf]

Suplementary Material

Examples Of Survey Questions

Do you think you should do stretching exercises?

○ Yes

○ No

If you think you should do stretching exercises...

Why? *(you can select multiple answers)*

☐ To avoid back pain

☐ To avoid joint pain

☐ To avoid muscle pain

☐ To avoid muscle stiffness

☐ To achieve greater range of motion

☐ Well-being

☐ Other (please specify)

What is the average duration of the stretching exercises you perform?

○ Less than 15 minutes

○ Between 15 and 30 minutes

○ Between 30 and 60 minutes

○ More than 60 minutes

How often have you done stretching exercises?

- ○ Every day
- ○ Every time I practise these sports
- ○ 1 to 5 times a week
- ○ Once or twice a month
- ○ 1 to 6 months per year
