# Supplementary material for: Beliefs, Barriers, and Stretching Practices Among Recreational Snowboarders and Alpine Skiers: A Cross-Sectional Study with a Generational Perspective
Source: Sports (Basel). 2026 Feb 3;14(2):55. doi: 10.3390/sports14020055 (PMC12944958; doi:10.3390/sports14020055)
Supplement: Supplementary file 1 [file sports-14-00055-s001.zip › Supplementary File S2.pdf]

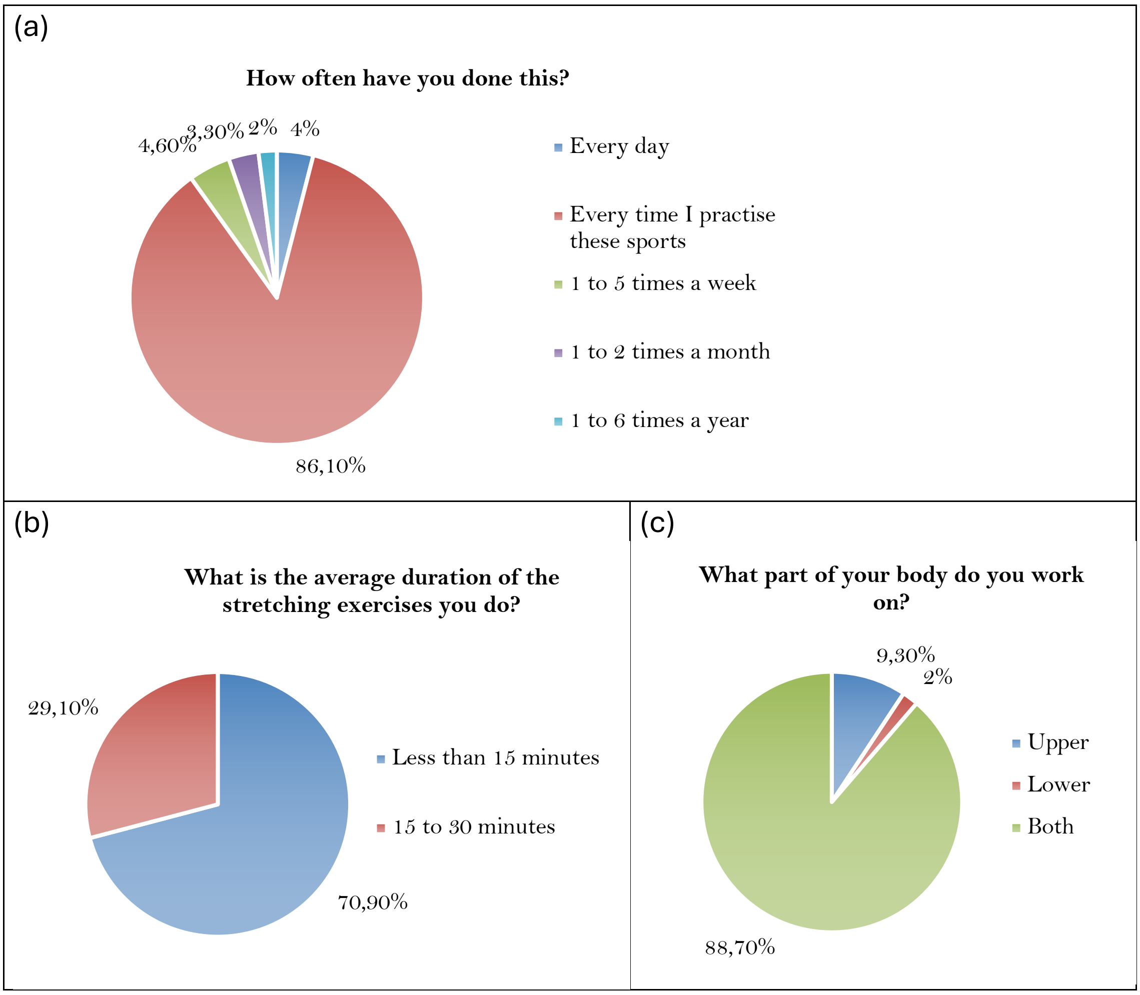

Figure S1. Descriptive characteristics of habitual stretching practices among recreational snowboarders and alpine skiers: (a) session duration, (b) frequency of performance, and (c) body regions targeted during stretching.
